# Supplementary material for: Empirical methods for controlling false positives and estimating confidence in ChIP-Seq peaks
Source: BMC Bioinformatics. 2008 Dec 5;9:523. doi: 10.1186/1471-2105-9-523 (PMC2628906; doi:10.1186/1471-2105-9-523)
Supplement: Additional File 1 — A variety of html documents from the USeq web site detailing the available applications, their best usage, output file type descriptions, command line menus, etc. [file 1471-2105-9-523-S1.zip › USeqUserGuides/JavaDoc.zip!/JavaDoc/util/bio/parsers/BlastCompompositeGenerator.html]

BlastCompompositeGenerator


|  |  |  |
| --- | --- | --- |
| **Overview** \| **Package** \| **Class** \| **Use** \| **Tree** \| **Deprecated** \| **Index** \| **Help** | |  |
| **PREV CLASS**   **NEXT CLASS** | **FRAMES**    **NO FRAMES**     **All Classes** |  |
| SUMMARY: NESTED \| FIELD \| CONSTR \| METHOD | DETAIL: FIELD \| CONSTR \| METHOD |  |


---


## util.bio.parsers Class BlastCompompositeGenerator

```
java.lang.Object
  util.bio.parsers.BlastCompompositeGenerator
```

---

``` public class BlastCompompositeGenerator extends java.lang.Object ```

---

**Constructor Summary**

`BlastCompompositeGenerator(java.lang.String[] args)`


| **Method Summary** | |
| --- | --- |
| `char[]` | `align(char[] q, char[] s)` |
| `static void` | `main(java.lang.String[] args)` |
| `void` | `makeBaseCounts()` |
| `void` | `scoreBlastResults()` |
| `void` | `scoreGroup(int bcIndex, char[] query, char[] align, char[] sbjct)` |
| `void` | `scoreLines(java.lang.String[] lines)` |

**Methods inherited from class java.lang.Object**

`equals, getClass, hashCode, notify, notifyAll, toString, wait, wait, wait`

**Constructor Detail**

### BlastCompompositeGenerator

```
public BlastCompompositeGenerator(java.lang.String[] args)
```


**Method Detail**

### scoreGroup

```
public void scoreGroup(int bcIndex,
                       char[] query,
                       char[] align,
                       char[] sbjct)
```

---


### align

```
public char[] align(char[] q,
                    char[] s)
```

---


### scoreLines

```
public void scoreLines(java.lang.String[] lines)
```

---


### scoreBlastResults

```
public void scoreBlastResults()
```

---


### makeBaseCounts

```
public void makeBaseCounts()
```

---


### main

```
public static void main(java.lang.String[] args)
```


---


|  |  |  |
| --- | --- | --- |
| **Overview** \| **Package** \| **Class** \| **Use** \| **Tree** \| **Deprecated** \| **Index** \| **Help** | |  |
| **PREV CLASS**   **NEXT CLASS** | **FRAMES**    **NO FRAMES**     **All Classes** |  |
| SUMMARY: NESTED \| FIELD \| CONSTR \| METHOD | DETAIL: FIELD \| CONSTR \| METHOD |  |


---
